# Aging under endocrine hormone regulation

**DOI:** 10.3389/fendo.2023.1223529

**Published:** 2023-08-02

**Authors:** Yutong Xing, Fan Xuan, Kaixi Wang, Huifeng Zhang

**Affiliations:** Second Hospital of Hebei Medical University, Shijiazhuang, China

**Keywords:** aging, endocrine, growth hormone, thyroid hormone, estrogen

## Abstract

Aging is a biological process in which the environment interacts with the body to cause a progressive decline in effective physiological function. Aging in the human body can lead to a dysfunction of the vital organ systems, resulting in the onset of age-related diseases, such as neurodegenerative and cardiovascular diseases, which can seriously affect an individual’s quality of life. The endocrine system acts on specific targets through hormones and related major functional factors in its pathways, which play biological roles in coordinating cellular interactions, metabolism, growth, and aging. Aging is the result of a combination of many pathological, physiological, and psychological processes, among which the endocrine system can achieve a bidirectional effect on the aging process by regulating the hormone levels in the body. In this paper, we explored the mechanisms of growth hormone, thyroid hormone, and estrogen in the aging process to provide a reference for the exploration of endocrine mechanisms related to aging.

## 1 Introduction

**Figure 1:**
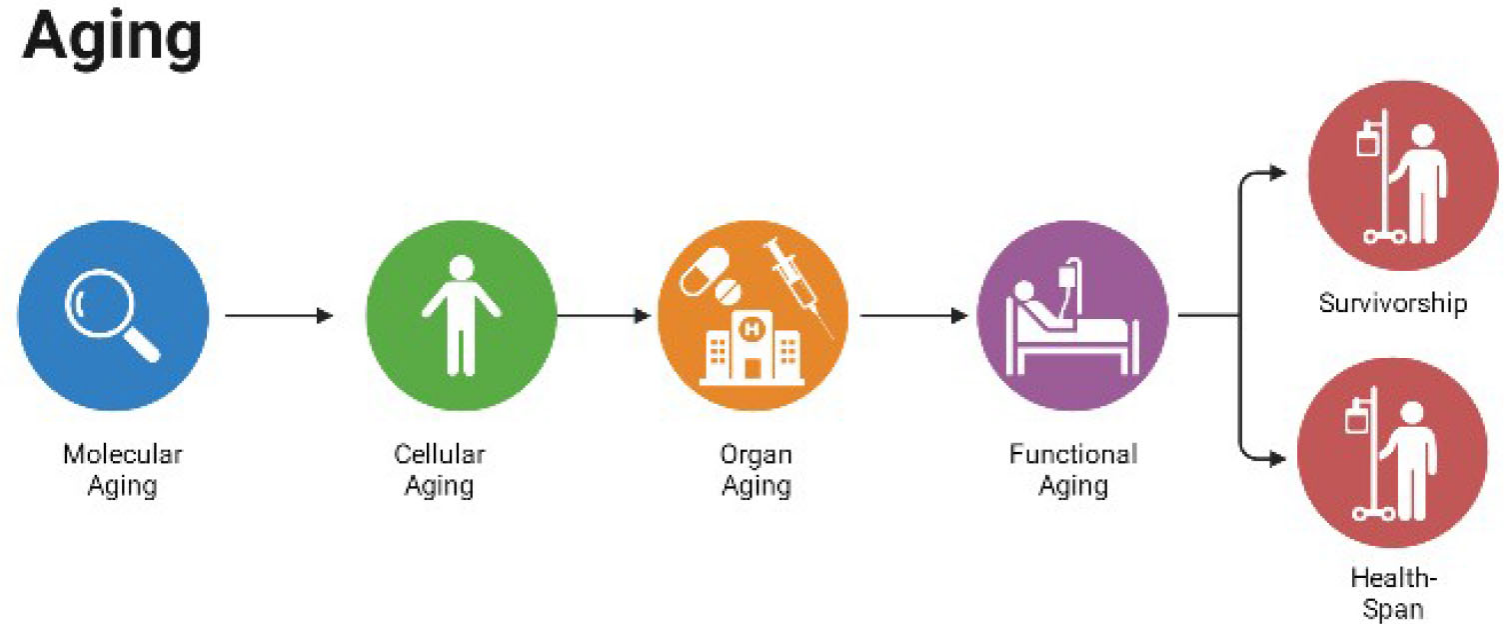
Aging.

Aging is a physiological process that involves a general decline in multiple physiological functions, leading to function loss and eventual death. Age-related changes in biological markers, such as oxygen free radicals (ROS), may arise from the damage and destruction of the body during aging, which may have implications for buffering, adjustment, and repair of aging. The endocrine system plays an important role in coordinating cellular interactions, metabolism, growth, and aging, and scholars have traditionally used endocrine hormone levels as a tool to induce, detect, and validate specific biological effects associated with aging. Recently, scholars have intensively explored the effects of endocrine hormones, such as growth hormone (GH), thyroid hormone (TH), and sex hormones, on the aging process and related diseases and death. Their findings suggest that the downstream pathways and genetic determinants of these related hormones currently dominate aging research, and genotyping the major functional factors of a particular endocrine pathway potentially can reveal the relationship between aging and emergence of a specific outcome. In this paper, we aimed to clarify the endocrine mechanisms associated with aging and their significance in maintaining a healthy life and increasing the life span by reviewing studies on aging and related endocrine hormones and their pathway major functional factors **Figure 1**.

### 1.1 Effect of GH on aging

#### 1.1.1 Overview of GH

GH is a peptide hormone synthesized, stored, and secreted by GH cells in the anterior pituitary gland. Its promotes protein synthesis and lipolysis in receptor cells of body tissues, organs, and bones, thus regulating body metabolism and growth and development. A series of physiological changes brought about by aging considerably affect an individual’s health. The aging process includes a reduction in GH, and mechanisms linking GH and aging include the evolutionarily conserved insulin/insulin-like growth factor (IGF-1) and rapamycin (mTOR) signaling pathways; these targets are consistent with trade-offs between metabolism, growth, and lifespan. The hepatocyte response to GH produces IGF-1, the primary active form of GH action, and increased IGF-I levels are associated with tumors, whereas decreased levels are associated with the development of various diseases, such as type 2 diabetes, hypoglycemia, osteoporosis, cognitive decline, and coronary artery disease, thus demonstrating the complexity of the role of the human IGF axis.

**Figure 2:**
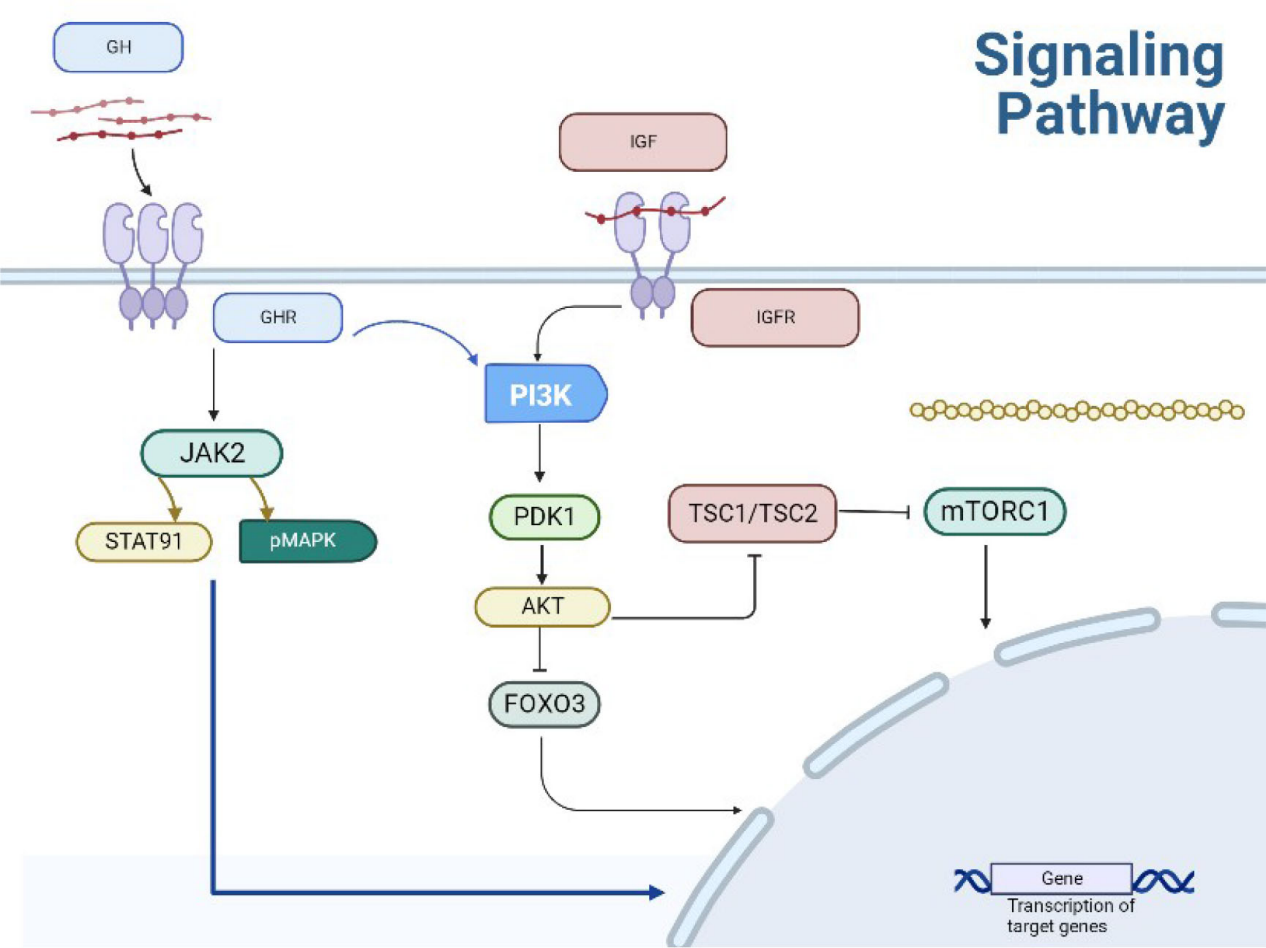
Transduction pathway of GH.

The intracellular signaling pathway of IGF-1 is the same as that induced by insulin. The IIS pathway is the most evolutionarily conserved pathway for aging control, with multiple targets including the FOXO family of transcription factors and mTOR complex. They are also involved in aging and remain conserved through evolution (1–3). Deregulated mTOR signaling is associated with cancer and diabetes progression as well as the aging process. The mechanisms linking GH signaling pathways to delayed aging and increased healthy lifespan are intertwined to form a complex network of interactions, including pleiotropic effects and regulatory loops (4). Animal studies have demonstrated that decreasing the activity of the GH/IGF-1/insulin system significantly increases lifespan. Compared with a control group, the offspring of centenarians exhibited relatively lower levels of cyclic IGF-I biological activity. There is an inverse relationship between IGF-I bioactivity and insulin sensitivity in the offspring of centenarians, indicating that IGF-I increases insulin sensitivity not only by inhibiting GH, but also by directly activating insulin receptors and IGF-IR. These data provide evidence for the involvement of the IGF-I/insulin system in regulating the aging process in humans (5) (**Figure 2**).

#### 1.1.2 Positive regulation of GH in aging and its role in promoting longevity

The amount of human GH secretion is closely related to age. Serum GH levels are high in the mid-fetus stage and at birth, and then decline rapidly within a few weeks to reach prepubertal levels by 6 months of age, and then peak at puberty. GH secretion after puberty is inversely proportional to age (6), with GH secretion decreasing gradually by approximately 15% every decade after the third decade of life. Therefore, some scholars suspect that reduced GH secretion is among the important causes of human aging. The effects of GH on the human body are not limited to promoting human growth and development; in fact, GH improved symptoms of atherosclerosis, enhanced bone density and bone remodeling, and reduced the risk of fracture (7). rhGH leads to increased muscle strength, fat metabolism, bone density, and skin thickness in middle-aged and elderly patients (6). Rosen et al. (8) found that rhGH also led to more energy, emotional stability, and sexual function in middle-aged and elderly people. In China, some scholars conducted rhGH treatment lasting up to 6 months in middle-aged and elderly men with menopausal symptoms, and the results showed that the participants’ physical performance, vasodilation, and psychosomatic symptoms were significantly improved as compared with those before treatment, suggesting that GH could effectively alleviate or improve some aging symptoms in middle-aged and elderly men, thus enhancing their quality of life. Moreover, mechanistically, Reyhan Westbroo et al. (9) found that GH slows down aging by modulating energy metabolism to increase fatty acid oxidation and mitochondrial efficiency. A recent study found that GH-treated aged mouse cells showed reduced reactive oxygen species (ROS) production and increased macrophage adhesion from aged mice to laminin and fibronectin substrates; in addition, cells obtained from aged mice exhibited increased migration as compared to young mice and a significant increase in macrophage migration was observed under GH stimulation (10). The discovery of these mechanisms provides a theoretical basis for the clinical application of GH in delaying aging.

#### 1.1.3 Negative regulation of GH in aging and its inhibitory role in longevity

Acromegaly due to GH overproduction due to anterior pituitary tumors is associated with an increased risk of hypertension, diabetes, and cancer and reduced life expectancy (11). In 1996, Brown-Borg et al. found that mice with hypopituitarism showed inability to secrete GH and lived longer than their counterparts. Studies in Brown-Borg’s laboratory showed that Ames dwarf mice produced less ROS, had higher activity of antioxidant enzymes (catalase, copper-zinc, and manganese superoxide dismutase) in the liver, kidney, heart, and hypothalamus, and were more sensitive to oxidative damage to proteins, lipids, and important nuclear and mitochondrial DNAs. Mitochondrial DNA caused less oxidative damage (12–14). Subsequent studies have shown that Ames pygmy mice are more resistant to paraquat, a toxic compound that produces substantial oxidative stress in the lungs and other organs, suggesting the biological significance of the enhanced antioxidant capacity of these animals (15, 16). Dermal fibroblasts isolated from Ames pygmy rats and other long-lived GH-related mutants were more resistant to various cytotoxic agents, glucose deprivation, and oxidative damage inducers in cultures compared to fibroblasts obtained from genetically normal controls (17). Studies conducted in the 1990s found that dwarf mice deficient in GH, prolactin, and thyroid-stimulating hormone (TSH) lived much longer than their normal siblings (18). The extended lifespan results in dwarf mice were thought to be largely due to GH deficiency (GHD), but this is now supported by a large body of evidence. Flurkey and his colleagues found that mice with GHD due to mutations in the gene encoding the GH-releasing hormone receptor also lived longer (19). The genetic condition of isolated GHD (IGHD) or GH resistance does not extend the human lifespan, but appears to extend healthy lifespan and provides strong protection of the organism against aging-related diseases in some cases. Kanfi et al. showed that mutant mice lacking SIRT6 exhibited an accelerated aging process (20), whereas SIRT6-overexpressing male transgenic mice lived longer than the control animals, which was associated with reduced serum IGF1 levels and other indicators of IGF-1 signaling. Genetic polymorphisms or mutations reducing the function of GH, IGF-1 receptor, insulin receptor, or downstream intracellular effectors, such as AKT, mTOR, and FOXO, were associated with longer lifespan in humans and model organisms. Multiple genetic manipulations that attenuate signal strength at different levels of the IIS pathway can sustainably extend lifespan in worms, flies, and mice (2). The hormones secreted by endocrine tissues in skeletal muscles have significant effects on muscle function, such as GH deficiency or glucocorticoid overdose and certain diseases. The psychological effects of GH are mainly achieved by stimulating the liver to produce IGF-1. GH therapy has been found to alleviate muscle atrophy caused by misuse. However, there is limited evidence regarding the impact of circulating IGF-1 on muscle development. IGF-1 plays a crucial role in regulating muscle mass through various mechanisms. These include stimulating muscle protein synthesis, inhibiting protein degradation, and facilitating the proliferation and fusion of satellite cells with existing muscle fibers. GH, specifically in skeletal muscle, stimulates the synthesis and growth of muscle cells, induces skeletal muscle DNA proliferation and protein synthesis *in vivo*, and downregulates myostatin, a key inhibitor of skeletal muscle regeneration. Moreover, GH reduces protein degradation. Notably, frail patients and elderly individuals with poor health in high-income countries exhibit significantly lower levels of GH (21).

IGF-1, due to its strong proliferative activity, acts as a significant risk factor for several tumors by regulating cell cycle, apoptosis, and cell survival. Long-lived individuals demonstrate higher insulin sensitivity and better preservation of beta cell function compared to younger patients. The endocrine and metabolic adaptations observed in centenarians may represent a physiological strategy to extend lifespan by slowing down cell growth and metabolism, thereby enhancing physiological reserve capacity and shifting cell metabolism from proliferation to repair activities, which helps in reducing the accumulation of senescent cells. These mechanisms are partly mediated by the regulation of the GH/IGF1/insulin system (22).

GH and IGF1 are important regulators of bone remodeling and metabolism, with important roles in achieving and maintaining bone mass throughout life. Evidence from animal models and human diseases suggests that GH overload results in an increased fracture risk in patients with GHD or acromegaly, which leads to organismal aging (23). In humans, abundant evidence demonstrates that an increased IGF-I level is a risk factor for several cancers. Therefore, since the discovery of IGF-IR in the 1980s, there has been great interest among researchers to develop drugs that inhibit IGF-IR signaling (e.g., tyrosine kinase inhibitors and IGF monoclonal antibodies) to treat various cancers. Recently, PTEN mice exhibit a general downregulation of the IIS pathway and increased energy expenditure, which is associated with improved mitochondrial oxidative metabolism and enhanced activity of brown adipose tissue. Similar to other mouse models with reduced IIS activity, PTEN-overexpressing and hypomorphic PI3K mice show an extended lifespan (24, 25). Paradoxically, GH and IGF-1 levels are decreased during normal aging, as well as in mouse models of premature aging (26). Thus, reduced IIS has been proposed as a common feature of both physiological aging and accelerated aging, whereas reduced structural IIS prolongs lifespan. Contrarily, a genetic analysis in extremely long-lived individuals revealed a higher incidence of IGF-IR gene mutations in female centenarians, as compared to controls. These mutations can cause functional alterations, and the lower adult lifetime height and higher serum free IGF-I levels in mutation carriers than in those without such mutations, whether this implies IGF-1 receptor insensitivity in this population, needs to be further investigated. Additionally, the IGF-IR level was lower in the transformed lymphoblastoid cells of IGF-IR mutation carriers, and the AKT activity induced by IGF-I was lower. The inhibition of histone demethylases (targeting H3K27) in worms may extend the lifespan by targeting components of key longevity pathways, such as the insulin/IGF-1 signaling pathway (27).

#### 1.1.4 Outlook

GH has a regulatory role in terms of endocrine aspects for healthy longevity, metabolism associated with aging, and so on; thus, we explore future hormonal treatments for human aging. Fahy et al. reported that treating people with GH in combination with dehydroepiandrosterone (a product of the adrenal cortex that serves as a precursor for sex hormone biosynthesis) and metformin (used to counteract the glucose-raising effects of GH) in healthy men aged 51–65 years induces protective immune changes and reduces epigenetic age to a rejuvenating level (28). GH has the potential to exert pro- and anti-aging effects at different stages of life. Primarily, this is reflected in the stimulation of growth, maturation, and fertility in early life, whereas, in later life, the effects of GH on body composition, immune function, and metabolism may be protective. In addition to its effects on the immune system, the lipolytic effects of GH may produce metabolic benefits, particularly in overweight or obese individuals, and GH-induced increases in muscle mass may help prevent and/or treat sarcopenia in the elderly.

### 1.2 Aging and THs

#### 1.2.1 Overview of THs

Further, TH is closely related to human growth, development, and energy metabolism, and its production is tightly regulated by the hypothalamic-pituitary-thyroid axis. The thyrotropin-releasing hormone (TRH) produced by the hypothalamus reaches the pituitary gland and binds to TRH receptors, stimulating the production and secretion of TSH, which subsequently binds to its receptor (TSHR) to induce TH production (29). Concurrently, TH inhibits the production and secretion of TRH and TSH in the hypothalamus and pituitary through the TH nuclear receptor β (THRβ), thus completing a typical negative feedback loop to maintain the physiological levels of TRH, TSH, and TH in the organism.

THs regulate metabolism-related molecular pathways in the body through protein interactions, such as PI3 K-akt-foxo1 and mTOR-p70S6 K signaling, to enhance oxygen consumption and ATP hydrolysis, reduce the mitochondrial coupling state, and induce catabolism of various energy sources (30). In turn, its receptor THR interacts with other nuclear hormone receptors, such as peroxisome proliferator-activated receptor and retinoic acid receptor, thus regulating metabolic pathways including lipids and sugars in different tissues (31). Meanwhile, recently, THRs has been found to regulate the expression of >80 genes involved in several metabolic processes, such as oxidative phosphorylation, tricarboxylic acid cycle, adipogenesis, and fatty acid catabolism (32). Thus, at the organismal level, THs, in addition to being necessary for maintaining growth and development, can increase basal metabolic rate and promote oxidative phosphorylation of substances, enhance cardiac systolic and diastolic functions, reduce peripheral vascular resistance, and lead to improved hemodynamics.

TH production undergoes dynamic changes with age. Compared with other control groups, healthy centenarians exhibited lower levels of serum TSH and FT3 and higher levels of serum rT3. FT3 levels and FT3/FT4 ratio decrease progressively with age, whereas FT4 and TSH increase with age. Among individuals aged 105 years and above (CENT/105+), higher FT4 levels and lower FT3/FT4 ratios are associated with impaired functional status and increased mortality. This indicates that age-related changes in TH function persist into advanced age, and the thyroid function in CENT/105+ individuals is highly diverse. The FT4 levels are negatively correlated with ADL score, whereas the FT3/FT4 ratio is positively correlated with ADL score. Additionally, FT3 levels and FT3/FT4 ratio are positively correlated with grip strength, whereas FT4 levels are negatively correlated with grip strength. FT4 levels are negatively correlated with SMMSE score, whereas TSH levels are positively correlated with SMMSE score. The FT3/FT4 ratio declines significantly with age, indicating a decrease in 5 ‘-deiodinase activity. It can be speculated that the increase in pro-inflammatory cytokines such as TNF-α, IL-1, and IL-6, which are characteristic of aging and longevity (inflammation), may inhibit 5’-deiodinase activity. The thyroid gland has limited adaptive strategies to meet metabolic and energy demands associated with age (22).

**Figure 3:**
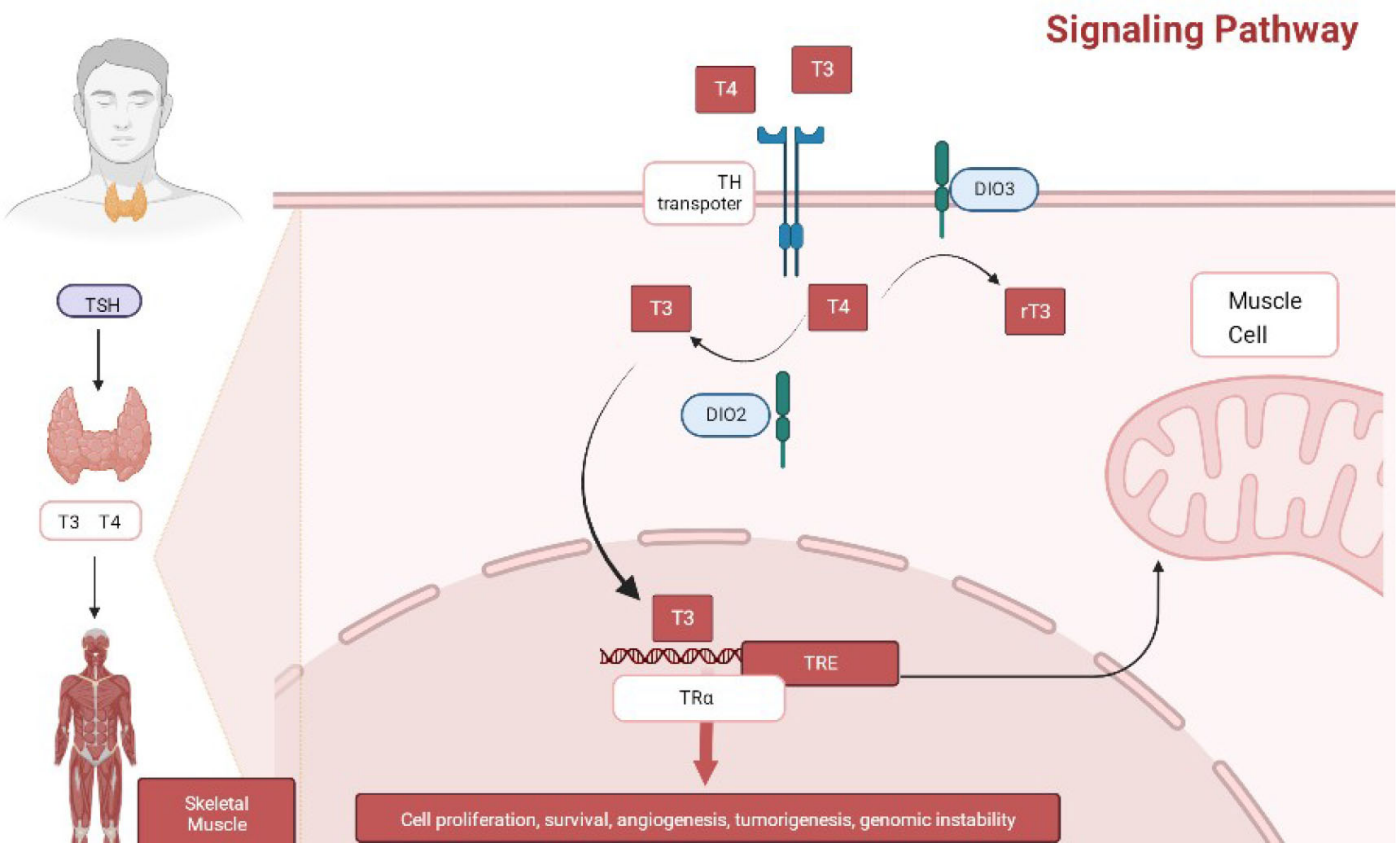
The signaling mechanism of TH.

In humans, hypothyroidism is associated with metabolic disorders, such as hypercholesterolemia and elevated low-density lipoprotein (LDL) levels, which increase the risk of developing diabetes mellitus and cardiovascular complications. Contrarily, subclinical hypothyroidism is associated with imbalances in bone metabolism, a predisposing factor of type 2 diabetes, hypertension, atrial fibrillation, and metabolic syndrome (33–35). Correspondingly, hyperthyroidism is associated with an increased risk of diabetes, dementia, neurocognitive dysfunctions, fractures, and cardiovascular-related diseases, which can lead to aging and even premature death (36–38). Additionally, both hyperthyroidism and hypothyroidism are associated with the development of certain types of diabetes and cancer (39, 40), and the molecular mechanisms of TH control are complex, with either too high or too low levels reducing the healthy life expectancy of people. Interventions based on the use of modulators of TH function may provide therapeutic benefits for some types of diabetes and cancer (41) (**Figure 3**).

#### 1.2.2 Regulation of THs in aging and their role in longevity

Recent anatomical studies have shown that the thyroid gland undergoes fibrosis, cellular infiltration, follicular changes, nodule formation, and weight loss as people age. The rate of iodine uptake by the thyroid gland tends to decrease progressively after 50 years of age. T3 and T4 syntheses are impaired due to a shortage of raw materials, with T3 synthesis being the most important factor. In this state, 5’deiodinase activity decreases, which reduces the availability of T4 in the surrounding tissues and reduces T3 clearance. Duntas et al. showed that, although T4 secretion was slightly reduced in the elderly, in some tissues, the ability to metabolize T4 by DIO1 and DIO2-mediated deiodination was reduced, thereby exhibiting normal T4 levels (42). Isolation studies in rodents have shown that the DIO1 activity is lower in older rats, liver levels of the TH transporter MCT8 are reduced, and TH receptor sensitivity changes with age (43). The physiological characteristics of TH-deficient mice (e.g., increased hepatic sensitivity to insulin, reduced fasting glucose level, reduced ROS production, enhanced antioxidant defense, enhanced resistance to oxidative stress, and reduced oxidative damage) appear to be highly beneficial in protecting tissues from potential damage, thereby delaying the aging process as compared to normal mice. Dwarf mice exhibit higher stress resistance through several metabolically induced genetic factors, such as Sirt1, FOXO, Klotho, and P66shc (44, 45). Higher serum TSH levels and/or lower serum free T4 levels are found to be associated with longer life expectancy, suggesting that TSH plays an important role in the aging process (29).

Early on, some scholars in China gave small oral doses of TH to healthy elderly people and found that the serum TH, IGF-1, and DHEA levels were significantly increased and the lipid levels were also reduced to varying degrees. Recent studies have found no significant effect of thyroxine replacement therapy in terms of improving cognitive function in elderly patients with subclinical hypothyroidism (46); Simonsick et al. proposed that high free T4 levels are a risk factor for poor prognosis and high mortality in cardiovascular disease (47); and Waring AC’s trial showed that low TSH levels or elevated free T4 levels were associated with population with reduced quality of life and increased risk of death (48). Accordingly, individuals with low free T4 or high TSH levels were expected to live 3.7 years longer than those with high free T4 or low TSH levels (43, 49). Zambrano et al. investigated the pro-aging effects of THs and found that active T3 bound to the THRβ isoform resulted in DNA damage and premature aging and was causally linked to the molecular process of accelerated aging (50). Additionally, the deleterious effects of chronic hyperthyroidism on life expectancy have been observed in mice and rats. Exposure to T4 resulted in a two-fold increase in serum T4 levels in wild-type mice, which had an approximately 50% reduction in the mean and maximum lifespan (51). These results provide theoretical support for the longevity benefits of hypothyroidism in the elderly.

Studies have shown that older people diagnosed with subclinical hypothyroidism at ≥70 years may have better physical function and social adaptation as compared with those with normal thyroid function at the same age (47). Similar studies have shown that lower free T4 levels are associated with better functional flexibility and physical performance in healthy normal thyroid populations between 68 and 97 years of age; hence, a lower free T4 level was proposed as a marker of healthy aging (47, 52). Additionally, Bano et al. reported the effect of TH level changes in hyperthyroid patients on gait speed in older adults, showing that higher TH levels were associated with slower gait speed (53, 54). Offspring of German-Jewish centenarians have higher TSH levels or lower circulating T3 or free T4 levels than offspring of non-centenarians (55). The Leiden Longevity Study also supports the association between low thyroid function and a lower risk of death from cardiovascular disease and longer life expectancy (56). The findings suggest that familial longevity is characterized by increased TSH secretion without changes in TH levels or energy metabolism (57, 58). Based on the Leiden study and other studies, high TSH levels are usually associated with healthy aging in the oldest age group (59). The fact that TSH and TH levels are not only associated with prolonged survival suggests that regulation of the negative feedback loop controlling TH production may contribute to this phenotype (58). Thus, the above experimental results are consistent with the viability theory of aging, suggesting that lower metabolic demands tend to favor longer healthy lifespans.

Despite the intricate nature of the aging process, its molecular foundation can be primarily attributed to a limited number of highly conserved mechanisms, which are responsible for the biological processes involved in maintaining and repairing the body. One unique characteristic observed in centenarians is a decline in TH levels. The molecular mechanisms underlying aging and chronic age-related diseases share common features, including inflammation, stress adaptation, protein imbalance, stem cell failure, metabolic disorders, and macromolecular damage. In middle-aged and elderly individuals without thyroid dysfunction, TH concentrations are positively associated with immune function indicators, such as complement protein levels, C-reactive protein levels, phagocyte activity, the percentage and count of natural killer cells and T cells, and the expression of activated monocytes. TH increases metabolic activity and oxygen consumption and induces oxidative stress in the short and long term. Elevated TH levels enhance the pro-inflammatory response of these cells, thus accelerating the aging process. Recent research suggests that the thyroid gland may play a role in the systemic accumulation of senescent cells. The continuous exposure of thyroid epithelial cells to ROS during the synthesis of THs through dioxidases can induce genomic damage and telomere erosion, which are indicators of cell senescence (60).

The direct effect of TH regulation on lifespan has recently been determined using PAX8 knockout mice and wild-type mice not treated with T4 (48). PAX8 is a major transcriptional regulator of thyroid organogenesis required for TH production. Using these mice, researchers determined the effects on health status and life expectancy in severely, mildly, and severely hyperthyroid mice as compared to control healthy mice. T4-treated hyperthyroid mice had decreased body weight, increased food intake, and reduced life expectancy (51). Direct regulation of TH levels using PAX8 heterozygous knockout mice, which suffered mild hypothyroidism due to direct thyroid defects but exhibited normal circulating levels of pituitary hormone alpha-GSU in adulthood, did not result in improved healthy lifespan or longer lifespan. Contrary to other experimental models of hypothyroidism (61, 62), PAX8 heterozygous mice realistically reflect the human hypothyroid phenotype, including insulin resistance, increased white adipose tissue mass, and increased triglyceride content in the skeletal muscle and liver (51). Similar to humans with hypothyroidism, these mice also exhibit reduced basal metabolic rate and obesity. Additionally, PAX8 heterozygous mice performed poorly in functional physical tests with accumulation of oxidative damage, suggesting that even minor alterations in TH levels (mild hypothyroidism) have profound effects on health breadth.

Studies on humans and animal models have shown a negative correlation between TH levels and lifespan. The multiple effects of reduced TH (including decreased metabolic rate, body temperature, and oxygen consumption, production of reactive oxygen clusters, and reduced oxidative damage) have important implications for longevity. TH signaling is involved in maintaining and integrating the body’s metabolic homeostasis at multiple levels and in processes, such as inflammatory responses and stem cell renewal; all of which contribute to the maintenance of homeostasis *in vivo*. Several recent studies have shown (56, 57, 63, 64) that higher serum TSH or lower serum free T4 levels are positively correlated with life expectancy in animals and humans, and that a slight decrease in TH function is associated with exceptional longevity. In healthy adults, higher serum FT4 levels (within the normal range) have been associated with lower physical performance scores and reduced grip strength. Some recent studies have reported that low levels of TSH are linked to an increased risk of dementia, implying that excessive production of FT4 could have detrimental effects on the central nervous system (22). At certain life stages, restriction of TH signaling favors optimal aging. However, studies directly targeting TH production have found that this does not extend the lifespan, suggesting that genetic or epigenetic traits are required to achieve maximum life expectancy in order to achieve longevity benefits (57).

#### 1.2.3 Outlook

Attempts have been made to slow down aging by reducing the production of damaging substances, e.g., ROS, due to metabolic activity. It has been proposed that moderate chronic caloric restriction may not only prolong lifespan but also delay age-related diseases in many species (both invertebrates and vertebrates) when nutritional status maintains essential life activities (65, 66). We should explore some other potential targets, such as lowering TH signaling that may help to reduce ROS. A previous study has suggested that hyperthyroidism leads to increased ROS production, which may be causally related to aging (67).

The main concept behind Thyroid Biographies is to systematically and accurately gather and store data that can be used to reconstruct a unique profile of lifelong variables for each individual or patient. These variables include factors such as age, gender, place of birth and residence, occupational history, socioeconomic and psychological status, lifestyle habits (such as diet and physical activity), medical conditions (with a specific focus on endocrine disorders), healthcare history (including parental medical history), coexisting diseases, results of comprehensive blood tests throughout life (including endocrine-related data), and medications used. All of these factors can have both, long-term and short-term effects on thyroid function and overall physiological well-being. Of particular importance are environmental factors, including exposure to substances that disrupt endocrine function, as well as habits like smoking and other conditions, which can influence the balance of thyroid function over a person’s lifetime (60).

TH is a potent regulator of lipid and glucose metabolism, reducing circulating levels of triglycerides and cholesterol-containing lipoproteins, and stimulates the expression of sterol response element binding protein 2 (Srebp2), thereby enhancing the hepatic cholesterol uptake. Dementia is a progressive deterioration in cognitive function caused by brain injury or diseases as the body ages and a combination of internal and external circumstances; dementia shows a much progressive deterioration than normal aging. Hypothyroidism with elevated TSH and reduced TH levels is a rare but reversible cause of cognitive dysfunction, and dementia in approximately 15%–20% of older adults is associated with TH levels (68). Large case-control studies have found an 81% increased risk of dementia in people aged ≥65 years with a history of hypothyroidism, with a more than three-fold increased risk of dementia in those with thyroid disease requiring TH replacement therapy. In 1997–1998, Abbey et al. (69) studied 3,075 independently living healthy volunteers aged 70–79 years in Memphis, Tennessee and Pittsburgh, Pennsylvania. A medication review and dementia diagnosis was conducted. The study included a 2-year follow-up of TSH level in patients not taking antithyroid medication, resulting in a higher probability of dementia with low TSH levels. None of the 23 participants with apparent hypothyroidism were diagnosed with dementia, which also provides evidence that dementia in aging is associated with higher TH levels. A subsample of 2089 individuals with TSH measurements was analyzed in the Mexican Health and Aging Study. Patients aged ≥50 years with subclinical hypothyroidism had a higher prevalence of geriatric syndrome as compared to patients with a normal thyroid function (70, 71).

### 1.3 Aging and estrogen

#### 1.3.1 Overview of estrogens

Estrogens are currently classified into three types, of which the most physiologically significant is estradiol (E2). Although E1 and E3 can be synthesized from estradiol, E2 has the highest affinity for the intracellular estrogen receptors ERα and ERβ. Moreover, E2 has a high affinity for the membrane-associated G protein-coupled receptor GPR30/GPER1 (72). The ovary is the main source of estrogen, which is synthesized from acetate and cholesterol in a multi-step process culminating in the use of cytochrome P450 aromatase. Aromatase is also expressed in non-ovarian sites, including the brain, testis, breast, and adrenal glands (73).

**Figure 4:**
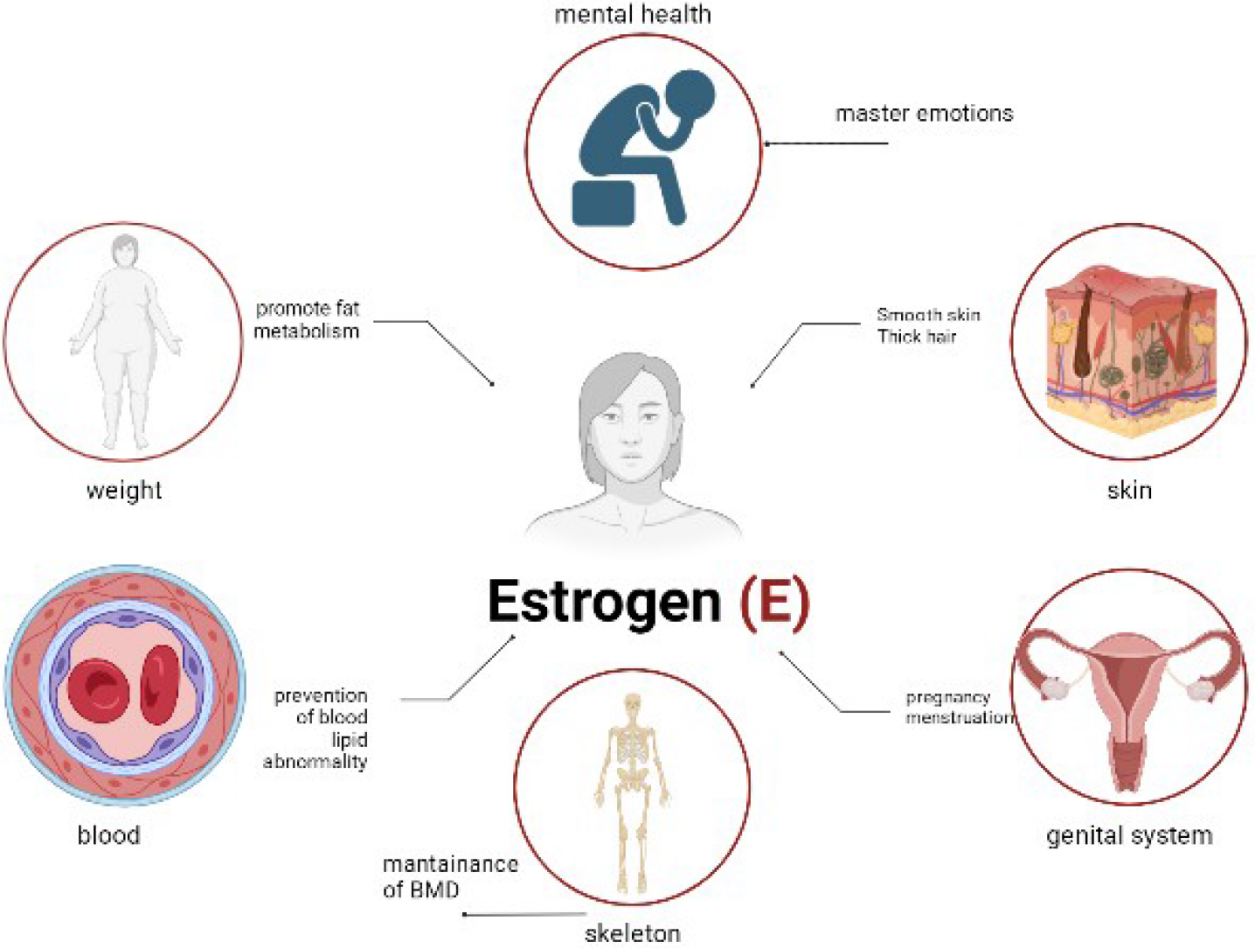
The physiological effects of estrogen.

Estrogen acts directly on the vessel wall *via* the estrogen receptor to enhance the production and release of endothelium-dependent relaxing factors or inhibit the activity of vasoconstrictive substances, thereby improving vascular endothelial function, which reduces coronary artery basal pressure, improves vasodilation, and prevents atherosclerosis formation. Studies have shown that 50%–80% of women experience vasodilatory symptoms, such as hot flushes and night sweats, at some point during the menopausal transition (74, 75). An increase in sleep disturbances has been reported during menopause, which may be due to secondary to vasodilatory symptoms (76). Physical symptoms, such as aches and pains, have also been reported through the menopausal transition (75). Various cognitive changes have been reported, including reduced processing speed and verbal memory observed during the perimenopausal period (77). Finally, depressive symptoms are also associated with menopause (75) (**Figure 4**).

#### 1.3.2 Regulation of estrogen in aging and its role in longevity

For women, menopause is the beginning of full-blown aging. Current research findings show that HRT is the most effective method of anti-aging treatment, and at the heart of HRT is estrogen. Estrogen action affects almost all tissues and is responsible for homeostatic regulation, cell proliferation and apoptosis, liver protein expression, lipid metabolism, energy balance, glucose metabolism, immune and cardiovascular changes, gonadotropin feedback and gametogenesis, brain neuronal development/memory processing and repair/neurodegeneration, and bone growth. risk factor for coronary artery disease. A meta-analysis (78) has shown that premenopausal and early menopausal estrogen supplementations have cardiovascular protective effects through improvement of lipid metabolism, mainly by increasing high-density lipoprotein levels, decreasing LDL levels, increasing apolipoprotein A levels, and decreasing apolipoprotein B levels. Castanho et al. (79) divided 94 postmenopausal women into no-treatment, estrogen, and estrogen groups. De Franciscis et al. (80) showed that 28 healthy menopausal women and 28 menopausal women with metabolic syndrome were treated with estrogen for 6 months. The results showed a significant reduction in total cholesterol and triglyceride levels and systolic blood pressure in the metabolic syndrome group after 6 months of treatment.

A dramatic decrease in estrogen levels leads to a concomitant decrease in bone mineral density. Epidemiological studies in humans and mechanistic studies in rodents suggest that an increase in ROS levels plays an important role in the decline in bone mineral density, which in turn leads to senescence, and that ROS greatly affects the production and survival of osteoclasts, osteoblasts, and osteocytes. Loss of estrogen or androgen reduces the bone defenses against oxidative stress, which explains the increased bone resorption associated with the acute loss of these hormones (81, 82). Consistent with the view that the beneficial effects of estrogen on bone are consistent with its antioxidant properties, the beneficial effects of estrogen on several other tissues, such as the heart, arteries, central nervous system, lens epithelium, fat, liver, and fallopian tubes, have also been shown to be due to improved defenses against ROS. During the 5 years of menopause, women rapidly lose large amounts of cancellous bone and slowly lose cortical and cancellous bones after 5 years (83, 84). Estrogen deficiency leads to an increase in blood interleukins 1, 6, and 7 and tumor necrosis factor, and the osteoclasts, which are produced in T cells, stromal cells, and osteoblasts, lead to an increase in the number of osteoclasts and a decrease in overall bone mineral density. Another study (85) found that estrogen maintains an appropriate ratio of osteoblast bone formation to osteoclast bone resorption by inducing apoptosis of pro-osteoblasts through the osteoblast paracrine signaling pathway.

E2 has both genomic and non-genomic effects. The non-genomic effect occurs through the activation of mitogen activated protein kinase (MAPK), which may be associated with synaptic plasticity in adults. Brain aging caused by estrogen deficiency manifests itself primarily as cognitive decline, including impairment of attention, memory, comprehension, judgment and, in the case of multiple cognitive domains, dementia. Age-related expression of estrogen receptors has been found in the brains of female animals in animal studies, and studies sampling from the cerebral cortex and hippocampus have shown a region-specific reduction in estrogen receptors (86). Hippocampus is particularly sensitive to fluctuations in estradiol due to the presence of aromatase in the human hippocampal region. Memory loss is more common during the menopausal transition. By using knockout animals, ERα, ERβ, and GPR30/GPER1 have been shown to mediate hippocampal synaptic plasticity and the cognitive effects of estrogen (87, 88). Selective ERα and subsequent ERβ activation is mediated through interaction with metabotropic glutamate receptors (89). Selective activation of ERα has been shown to increase dendritic spine density and cognitive abilities (90). GPR30/GPER1 has been shown to induce synaptic plasticity in the hippocampus following selective activation. This suggests that ERα, ERβ, and GPR30/GPER1 all regulate cognition. Age-related reductions in sex hormones are risk factors for Alzheimer’s disease (91). Jacobs et al. (92) found that the leukocyte telomere length was significantly shorter in the ApoE-ϵ4 carrier group than in the no-Alzheimer’s disease group. The shorter telomere lengths resulted in faster cellular senescence, whereas estrogen treatment of the ApoE-ϵ4 carrier group for approximately 2 years delayed cellular senescence and hence the onset of dementia. In menopausal women, hormone therapy is beneficial in terms of verbal memory and executive function, thereby delaying the onset of dementia (93). In addition to the classical estrogen receptors ERα and ERβ and GPCR GPR30/GPER1, there is evidence for two additional membrane-localized estrogen receptors in the central nervous system (94). In rodents, ovariectomy results in a reduction in dendritic spines, most notably in apical CA1 pyramidal cells, which can be restored with hormone replacement therapy (95). In rhesus monkeys, natural menopause promotes the selective loss of specific synaptic classes of perforating synapses in the outer molecular layer of the dentate gyrus. These synapses are thought to be important in maintaining synaptic enhancement over long time courses (96). The same group subsequently found that E2 treatment increased the synaptic density in the prefrontal cortex of overly resected older rhesus monkeys (97), whereas E2 treatment in rescued older rhesus monkeys from ovariectomy had reduced dorsolateral polysynaptic buttons in the prefrontal cortex (98).

Additionally, estrogen receptors have been found in the epithelial tissue of the bladder, bladder triangle, urethra, vaginal mucosa, uterosacral ligament, anal raphe, and pubocervical fascia, and estrogen increases the epithelial maturation index in these tissues and reduces genital tract atrophy and hypersecretion, thereby improving sexual function (99).

#### 1.3.3 Clinical use of estrogen in anti-aging

Long-term estrogen treatment prevents the activation of apoptosis signal-regulated kinase 1 and its downstream effectors JNK and p38 MAPK. Estrogen-treated cardiomyocytes were more resistant to angiotensin II-induced apoptosis; the anti-apoptotic and cardioprotective effects of E2 were blocked by ER antagonists and Trx reductase inhibitors (dulcolic acid), suggesting that long-term estrogen treatment improves congestive heart failure through antioxidant mechanisms (100).

During the aging process, especially after menopause, estrogen-deficient skin can lead to a dramatic decline in skin health. For example, postmenopausal women not receiving hormone replacement therapy experience a 2% decrease in collagen content per year of menopause, and the lack of estrogen exacerbates aging. Aging skin leads to breakage of collagen and elastin fibers through the action of matrix metalloproteinases, and increased mitochondrial oxidative stress leads to mitochondrial DNA deficiency in dermal fibroblasts. Skin blood flow and lipid deposition decrease with subcutaneous aging (101, 102). Postmenopausal estrogen therapy (ET) has been the mainstay of treatment for postmenopausal women and involves maintaining healthy skin and progestins (e.g., progesterone) blocking the expression of estrogen receptors in the uterus, thereby avoiding endometrial activation during ET. The combination of ET and progesterone is known as menopausal hormone therapy.

### 1.4 Regulation of GH, TH, and estrogen in aging and their interactions in longevity

GH stimulates growth, cellular reproduction, and regeneration in humans and other animal species primarily by stimulating the production of IGF-I, which in turn exerts negative feedback on the pituitary and hypothalamus, while circulating GH levels inhibit central GH production. Additionally, TH increases GH gene expression and secretion through its actions on the hypothalamus and pituitary gland in rats and humans (103). The link between growth and longevity is well established in animal models, such as GHR knockout (KO) mice, which have reduced GH activity and the highest lifespan of any laboratory mouse model. Additionally, the removal of the pituitary gland, which eliminates the secretion of endocrine signals such as GH, TSH, and prolactin, prolongs lifespan (104). Other studies have shown that, in a group of young mice, body weight was negatively correlated with IGF-I, leptin, and THs. Whereas the results in these animal studies may not be a good fit to guide hormone therapy in humans, the relationship between the growth tropism axis and healthy lifespan in humans is more controversial.

Both elevated TH elevated IGF-1 in the circulation and increased IGF-1 bioavailability. Contrarily, in Laron (GH receptor knockout), Ames (prop1 mutation), and Snell (pit1 mutation) dwarf mice, those mice that survived the longest exhibited a severely reduced thyroid function (105). These mouse models show a healthy aging phenotype, including hypothyroidism, preservation of neurocognitive and muscle function, reduced cancer incidence, enhanced insulin response, and improved glucose tolerance (104, 106). Contrarily, at the molecular level, these mice exhibit reduced signaling *via* the insulin and IGF-1 pathways, which results in restricted phosphorylation of downstream targets, such as serum/glucocorticoid-regulated kinases and AKT. The restricted activity of these kinases promotes the entry of FOXO transcription factor into the nucleus where it regulates transcription of genes that promote longevity (107).

In mammalian models, many of the effects of GH are mediated through IGF-I. As with GH, there are many pathways of interaction between IGF-I and TH. The role of T3 at the hypothalamic and pituitary levels drives IGF-I mRNA production. TH may have a role in determining lifespan that is not yet fully understood. However, the questions on the primary and dependent variables in the many pathways still remain.

The results of Fahy et al.’s study (108) showed that treatment among healthy men aged 51-65 years with GH in combination with dehydroepiandrosterone (a precursor of sex hormone biosynthesis) and metformin induced protective immune changes, restored thymic function, and reduced epigenetic age to a level suggestive of regeneration. It also suggests that GH may have both pro- and anti-aging effects at different stages of life.

Many age-related diseases involve dysregulation of endocrine hormones. Let’s take sarcopenia as an example. Sarcopenia refers to inadequate muscle mass and low grip strength, with low grip strength alone being defined as probable sarcopenia. The diagnosis of sarcopenia is based on identifying low muscle mass or muscle strength. Severe sarcopenia is indicated by additional signs of poor physical performance. According to the original definition by the European Working Group on Sarcopenia in Older People, the presence of reduced muscle mass without reduced muscle function can be categorized as pre-sarcopenia. The development of sarcopenia involves various factors, including acute or chronic illness, chronic inflammation, reduced calorie intake, and immobility. Muscle and bone, as secretory organs, play a role in regulating body metabolism through autocrine, paracrine, or endocrine effects. They interact through myokines, which are a variety of myogenic effectors.

In aging muscle, GCs stimulate the ubiquitin-proteasome and lysosomal systems, affecting anabolic muscle growth factors (including IGF-1) and negative growth regulators (such as myostatin), resulting in decreased protein synthesis. Local metabolism of glucocorticoids in muscles, such as the activity of 11β-hydroxysteroid dehydrogenase type 1 (11β-HSD1), is associated with muscle atrophy and sarcopenia. In the aging HPA axis, glucocorticoids promote muscle atrophy. Sex hormones also play a role in senile sarcopenia, and hormone replacement therapy is a promising avenue for its treatment and prevention. Hyperthyroidism can manifest as thyrotoxic myopathy, characterized by muscle weakness, tissue damage, and elevated creatine kinase (CK) levels. Effective treatment can restore muscle function and normalize CK measurements. Vitamin D deficiency is associated with an increased risk of reduced muscle strength and physical function in postmenopausal women. The vitamin D randomized controlled trial in elderly participants revealed that baseline plasma vitamin D concentrations below 25 nmol/L were linked to reduced grip strength.

Dietary interventions, such as caloric restriction, increased protein intake, and vitamin D supplementation, have shown potential in the relationship between sarcopenic obesity and androgen deficiency in aged male C57BL6 mice. Hormonal changes are part of a complex system, and a comprehensive approach that considers physical activity, nutrition, and overall health is recommended. Resistance exercise, adequate protein intake, and vitamin supplementation are important considerations (21).

### 1.5 Artificial intelligence and aging

Aging is a fundamental characteristic shared by all living organisms, tissues, and cells. Artificial intelligence (AI) can leverage large medical databases collected over many years by companies and healthcare providers to extract crucial features, identify biological targets, and unveil underlying mechanisms. Through the use of generative adversarial networks (GANs) and reinforcement learning (RL), AI can uncover relevant patterns and discover new biological targets in complex non-linear data. This enables the generation of personalized medicines tailored to specific properties that hold clinical value in the healthcare industry. The application of AI algorithms in aging research presents significant opportunities, spanning various aspects such as prediction and treatment of aging-related conditions. Recurrent neural networks, GANs, and transfer learning techniques are gaining popularity in aging research for their ability to address diverse healthcare applications. These technologies aim to expedite the development of novel drugs in a faster, more cost-effective manner. AI can also enhance imaging and laboratory science by accelerating the identification of age-related biomarkers (109, 110). By reducing error rates and predicting treatment outcomes, including small molecule therapy and gene therapy, AI can improve patient outcomes (111). Furthermore, as AI technologies advance, they hold the potential to redefine mental disorders more objectively than the current DSM-5 criteria. This includes early identification or detection at precursor stages of mental disorders, enabling interventions that are more effective and personalized based on an individual’s unique characteristics (112).

## 2 Outlook

Aging is characterized by a progressive loss of physiological integrity, and this change is a major risk factor for major human pathological diseases. Aging is accompanied by a progressive loss of anatomical structures and physiological functions of the body (organs, tissues, and cells), leading to age-related diseases, such as cardiovascular diseases (atherosclerosis, stroke, and so on), musculoskeletal disorders (sarcopenia and osteoporosis), neurodegenerative diseases (dementia and so on), immune disorders (arthritis and inflammation), endocrine/metabolic disorders (diet, obesity, insulin resistance, and so on),gastrointestinal disorders (irritable bowel, celiac disease, and so on) and various cancers. Recently, the rate of aging is controlled, at least in part, by evolutionarily conserved genetic pathways and biochemical processes. The common features of aging in different organisms include genomic instability, telomere wear, altered epigenetics, loss of protein homeostasis, dysregulated nutrient perception, mitochondrial dysfunction, cellular senescence, stem cell depletion, and altered intercellular communication. The accumulation of cellular damage over time is widely recognized as a general cause of aging. Since the incidence of age-related diseases increases logarithmically with age, a deeper understanding of the biological processes of aging could help to elucidate pathophysiological changes in the elderly and possibly reduce the incidence of age-related diseases.

The aging process is accompanied by the development or aggravation of underlying diseases and the reduction of healthy age. Therefore, we have tried to summarize previous studies to analyze the relationship between endocrine hormones and aging to guide clinical interventions for delaying aging.

In conclusion, recent research has revealed that the endocrine system has a bidirectional effect on the regulation of aging through the regulation of hormone levels in the body. As mentioned above, GH, TH, and estrogen can all have positive or negative targeting effects on different tissues, organs, or systems in the body through specific molecular pathways to accelerate or retard the aging process. However, most of these findings have not yet been put into clinical use, and the exploration of their effects on human aging is still at the basic research stage. Therefore, there is a greater need to combine basic research findings with clinical applicability to explore the benefits and harms of hormone replacement therapy or hormone inactivation therapy on the body as a whole, to slow down the aging process and achieve an increase in the average lifespan in all human beings.

## Author contributions

YX contributed to conception. KW and YX wrote the original manuscript. FX contributed to review of the manuscript. HZ designed the project and edited the final manuscript. All authors contributed to the article and approved the submitted version.

## References

[1] Barzilai N Huffman DM Muzumdar RH Bartke A. The critical role of metabolic pathways in aging. Diabetes (2012) 61(6):1315–22. doi: 10.2337/db11-1300 PMC3357299 22618766

[2] Fontana L Partridge L Longo VD. Extending healthy life span–from yeast to humans. Science (2010) 328(5976):321–6. doi: 10.1126/science.1172539 PMC3607354 20395504

[3] Kenyon CJ. The genetics of ageing. Nature (2010) 464(7288):504–12. doi: 10.1038/nature08980 20336132

[4] Kim YC Guan KL. mTOR: a pharmacologic target for autophagy regulation. J Clin Invest (2015) 125(1):25–32. doi: 10.1172/JCI73939 25654547 PMC4382265

[5] Vitale G Brugts MP Ogliari G Castaldi D Fatti LM Varewijck AJ. Low circulating IGF-I bioactivity is associated with human longevity: findings in centenarians’ offspring. Aging (Albany NY) (2012) 4(9):580–9. doi: 10.18632/aging.100484 PMC3492223 22983440

[6] Rudman D Feller AG Nagraj HS Gergans GA Lalitha PY Goldberg AF. Effects of human growth hormone in men over 60 years old. N Engl J Med (1990) 323(1):1–6. doi: 10.1056/NEJM199007053230101 2355952

[7] Simpson H Savine R Sönksen P Bengtsson B-A Carlsson L Christiansen JS. Growth hormone replacement therapy for adults: into the new millennium. Growth Horm IGF Res (2002) 12(1):1–33. doi: 10.1054/ghir.2001.0263 12127299

[8] Rosén T Wirén L Wilhelmsen L Wiklund I Bengtsson BA. Decreased psychological well-being in adult patients with growth hormone deficiency. Clin Endocrinol (Oxf) (1994) 40(1):111–6. doi: 10.1111/j.1365-2265.1994.tb02452.x 8306469

[9] Westbrook R Bonkowski MS Strader AD Bartke A. Alterations in oxygen consumption, respiratory quotient, and heat production in long-lived GHRKO and Ames dwarf mice, and short-lived bGH transgenic mice. J Gerontol A Biol Sci Med Sci (2009) 64(4):443–51. doi: 10.1093/gerona/gln075 PMC2657169 19286975

[10] Cruz Borbely KS Marques A Porto FL Mendonça BS Smaniotto S Dos Santos Reis MD. Growth hormone stimulates murine macrophage migration during aging. Curr Aging Sci (2022) 15(3):266–73. doi: 10.2174/1874609815666220415132815 35430985

[11] Mercado M Gonzalez B Vargas G Ramirez C Espinosa de los Monteros AL Sosa E. Successful mortality reduction and control of comorbidities in patients with acromegaly followed at a highly specialized multidisciplinary clinic. J Clin Endocrinol Metab (2014) 99(12):4438–46. doi: 10.1210/jc.2014-2670 25210882

[12] Brown-Borg HM Rakoczy SG Romanick MA Kennedy MA. Effects of growth hormone and insulin-like growth factor-1 on hepatocyte antioxidative enzymes. Exp Biol Med (Maywood) (2002) 227(2):94–104. doi: 10.1177/153537020222700203 11815672

[13] Brown-Borg HM Rakoczy SG. Catalase expression in delayed and premature aging mouse models. Exp Gerontol (2000) 35(2):199–212. doi: 10.1016/S0531-5565(00)00079-6 10767579

[14] Brown-Borg HM Bode AM Bartke A. Antioxidative mechanisms and plasma growth hormone levels: potential relationship in the aging process. Endocrine (1999) 11(1):41–8. doi: 10.1385/ENDO:11:1:41 10668640

[15] Panici JA Harper JM Miller RA Bartke A Spong A Masternak MM. Early life growth hormone treatment shortens longevity and decreases cellular stress resistance in long-lived mutant mice. FASEB J (2010) 24(12):5073–9. doi: 10.1096/fj.10-163253 PMC2992365 20720157

[16] Bokov AF Lindsey ML Khodr C Sabia MR Richardson A. Long-lived ames dwarf mice are resistant to chemical stressors. J Gerontol A Biol Sci Med Sci (2009) 64(8):819–27. doi: 10.1093/gerona/glp052 PMC2981464 19414510

[17] Harrison BC Bell ML Allen DL Byrnes WC Leinwand LA. Skeletal muscle adaptations in response to voluntary wheel running in myosin heavy chain null mice. J Appl Physiol (1985) . 2002. 92(1):313–22. doi: 10.1152/japplphysiol.00832.2001 11744674

[18] Brown-Borg HM Borg KE Meliska CJ Bartke A. Dwarf mice and the ageing process. Nature (1996) 384(6604):33. doi: 10.1038/384033a0 8900272

[19] Flurkey K Papaconstantinou J Miller RA Harrison DE. Lifespan extension and delayed immune and collagen aging in mutant mice with defects in growth hormone production. Proc Natl Acad Sci U S A (2001) 98(12):6736–41. doi: 10.1073/pnas.111158898 PMC34422 11371619

[20] Mostoslavsky R Chua KF Lombard DB Pang WW Fischer MR Gellon L. Genomic instability and aging-like phenotype in the absence of mammalian SIRT6. Cell (2006) 124(2):315–29. doi: 10.1016/j.cell.2005.11.044 16439206

[21] Kamwa V Welch C Hassan-Smith ZK. The endocrinology of sarcopenia and frailty. Minerva Endocrinol (Torino) (2021) 46(4):453–68. doi: 10.23736/S2724-6507.20.03198-3 33331737

[22] Ostan R Monti D Mari D Arosio B Gentilini D Ferri E. Heterogeneity of thyroid function and impact of peripheral thyroxine deiodination in centenarians and semi-supercentenarians: association with functional status and mortality. J Gerontol A Biol Sci Med Sci (2019) 74(6):802–10. doi: 10.1093/gerona/gly194 30165411

[23] Mazziotti G Lania AG Canalis E. Skeletal disorders associated with the growth hormone-insulin-like growth factor 1 axis. Nat Rev Endocrinol (2022) 18(6):353–65. doi: 10.1038/s41574-022-00649-8 35288658

[24] Ortega-Molina A Efeyan A Lopez-Guadamillas E Muñoz-Martin M Gómez-López G Cañamero M. Pten positively regulates brown adipose function, energy expenditure, and longevity. Cell Metab (2012) 15(3):382–94. doi: 10.1016/j.cmet.2012.02.001 22405073

[25] Foukas LC Bilanges B Bettedi L Pearce W Ali K Sancho S. Long-term p110α PI3K inactivation exerts a beneficial effect on metabolism. EMBO Mol Med (2013) 5(4):563–71. doi: 10.1002/emmm.201201953 PMC3628103 23483710

[26] Schumacher B van der Pluijm I Moorhouse MJ Kosteas T Robinson AR Suh Y. Delayed and accelerated aging share common longevity assurance mechanisms. PloS Genet (2008) 4(8):e1000161. doi: 10.1371/journal.pgen.1000161 18704162 PMC2493043

[27] Kim EB Fang X Fushan AA Huang Z Lobanov AV Han L. Genome sequencing reveals insights into physiology and longevity of the naked mole rat. Nature (2011) 479(7372):223–7. doi: 10.1038/nature10533 PMC3319411 21993625

[28] Bartke A Hascup E Hascup K Masternak MM. Growth hormone and aging: new findings. World J Mens Health (2021) 39(3):454–65. doi: 10.5534/wjmh.200201 PMC8255405 33663025

[29] Liu YC Yeh CT Lin KH. Molecular functions of thyroid hormone signaling in regulation of cancer progression and anti-apoptosis. Int J Mol Sci (2019) 20(20):1–17. doi: 10.3390/ijms20204986 PMC6834155 31600974

[30] Johannsen DL Galgani JE Johannsen NM Zhang Z Covington JD Ravussin E. Effect of short-term thyroxine administration on energy metabolism and mitochondrial efficiency in humans. PloS One (2012) 7(7):e40837. doi: 10.1371/journal.pone.0040837 22844412 PMC3406028

[31] Kouidhi S Clerget-Froidevaux MS. Integrating thyroid hormone signaling in hypothalamic control of metabolism: crosstalk between nuclear receptors. Int J Mol Sci (2018) 19(7):1–12. doi: 10.3390/ijms19072017 PMC6073315 29997323

[32] Singh BK Sinha RA Tripathi M Mendoza A Ohba K Sy JAC. Thyroid hormone receptor and ERRα coordinately regulate mitochondrial fission, mitophagy, biogenesis, and function. Sci Signal (2018) 11(536):2–12. doi: 10.1126/scisignal.aam5855 29945885

[33] Gao N Zhang W Zhang YZ Yang Q Chen SH. Carotid intima-media thickness in patients with subclinical hypothyroidism: a meta-analysis. Atherosclerosis (2013) 227(1):18–25. doi: 10.1016/j.atherosclerosis.2012.10.070 23159101

[34] Taylor PN Razvi S Pearce SH Dayan CM. Clinical review: A review of the clinical consequences of variation in thyroid function within the reference range. J Clin Endocrinol Metab (2013) 98(9):3562–71. doi: 10.1210/jc.2013-1315 23824418

[35] Wang F Tan Y Wang C Zhang X Zhao Y Song X. Thyroid-stimulating hormone levels within the reference range are associated with serum lipid profiles independent of thyroid hormones. J Clin Endocrinol Metab (2012) 97(8):2724–31. doi: 10.1210/jc.2012-1133 22730515

[36] Brandt F Thvilum M Almind D Christensen K Green A Hegedüs L. Morbidity before and after the diagnosis of hyperthyroidism: a nationwide register-based study. PloS One (2013) 8(6):e66711. doi: 10.1371/journal.pone.0066711 23818961 PMC3688572

[37] Kim HJ Kang T Kang MJ Ahn HS Sohn SY. Incidence and mortality of myocardial infarction and stroke in patients with hyperthyroidism: A nationwide cohort study in Korea. Thyroid (2020) 30(7):955–65. doi: 10.1089/thy.2019.0543 32093587

[38] Aubert CE Bauer DC da Costa BR Feller M Rieben C Simonsick EM. The association between subclinical thyroid dysfunction and dementia: The Health, Aging and Body Composition (Health ABC) Study. Clin Endocrinol (Oxf) (2017) 87(5):617–26. doi: 10.1111/cen.13458 PMC5658241 28850708

[39] López-Noriega L Cobo-Vuilleumier N Narbona-Pérez ÁJ Araujo-Garrido JL Lorenzo PI Mellado-Gil JM. Levothyroxine enhances glucose clearance and blunts the onset of experimental type 1 diabetes mellitus in mice. Br J Pharmacol (2017) 174(21):3795–810. doi: 10.1111/bph.13975 PMC5647183 28800677

[40] Zhang GY Langan EA Meier NT Funk W Siemers F Paus R. Thyroxine (T4) may promote re-epithelialisation and angiogenesis in wounded human skin ex vivo. PloS One (2019) 14(3):e0212659. doi: 10.1371/journal.pone.0212659 30925152 PMC6440638

[41] Gauthier BR Sola-García A Cáliz-Molina MÁ Lorenzo PI Cobo-Vuilleumier N Capilla-González V. Thyroid hormones in diabetes, cancer, and aging. Aging Cell (2020) 19(11):e13260. doi: 10.1111/acel.13260 33048427 PMC7681062

[42] Duntas LH. Thyroid disease and lipids. Thyroid (2002) 12(4):287–93. doi: 10.1089/10507250252949405 12034052

[43] Chaker L Cappola AR Mooijaart SP Peeters RP. Clinical aspects of thyroid function during ageing. Lancet Diabetes Endocrinol (2018) 6(9):733–42. doi: 10.1016/S2213-8587(18)30028-7 30017801

[44] Migliaccio E Giorgio M Mele S Pelicci G Reboldi P Pandolfi PP. The p66shc adaptor protein controls oxidative stress response and life span in mammals. Nature (1999) 402(6759):309–13. doi: 10.1038/46311 10580504

[45] Kurosu H Yamamoto M Clark JD Pastor JV Nandi A Gurnani P. Suppression of aging in mice by the hormone Klotho. Science (2005) 309(5742):1829–33. doi: 10.1126/science.1112766 PMC2536606 16123266

[46] Park YJ Lee EJ Lee YJ Choi SH Park JH Lee SB. Subclinical hypothyroidism (SCH) is not associated with metabolic derangement, cognitive impairment, depression or poor quality of life (QoL) in elderly subjects. Arch Gerontol Geriatr (2010) 50(3):e68–73. doi: 10.1016/j.archger.2009.05.015 19545916

[47] Simonsick EM Chia CW Mammen JS Egan JM Ferrucci L. Free thyroxine and functional mobility, fitness, and fatigue in euthyroid older men and women in the baltimore longitudinal study of aging. J Gerontol A Biol Sci Med Sci (2016) 71(7):961–7. doi: 10.1093/gerona/glv226 PMC4906324 26791089

[48] Waring AC Arnold AM Newman AB Bùzková P Hirsch C Cappola AR. Longitudinal changes in thyroid function in the oldest old and survival: the cardiovascular health study all-stars study. J Clin Endocrinol Metab (2012) 97(11):3944–50. doi: 10.1210/jc.2012-2481 PMC3485600 22879629

[49] Bano A Chaker L Mattace-Raso F Terzikhan N Kavousi M Ikram MA. Thyroid function and life expectancy with and without noncommunicable diseases: A population-based study. PloS Med (2019) 16(10):e1002957. doi: 10.1371/journal.pmed.1002957 31652264 PMC6814213

[50] Zambrano A García-Carpizo V Gallardo ME Villamuera R Gómez-Ferrería MA Pascual A. The thyroid hormone receptor β induces DNA damage and premature senescence. J Cell Biol (2014) 204(1):129–46. doi: 10.1083/jcb.201305084 PMC3882795 24395638

[51] López-Noriega L Capilla-González V Cobo-Vuilleumier N Martin-Vazquez E Lorenzo PI Martinez-Force E. Inadequate control of thyroid hormones sensitizes to hepatocarcinogenesis and unhealthy aging. Aging (Albany NY) (2019) 11(18):7746–79. doi: 10.18632/aging.102285 PMC6781991 31518338

[52] Selmer C Olesen JB Hansen ML. Subclinical and overt thyroid dysfunction and risk of all-cause mortality and cardiovascular events: a large population study. J Clin Endocrinol Metab (2014) 99(7):2372–82. doi: 10.1210/jc.2013-4184 24654753

[53] Bano A Chaker L Darweesh SK Korevaar TIM Mattace-Raso FUS Dehghan A. Gait patterns associated with thyroid function: The Rotterdam Study. Sci Rep (2016) 6:38912. doi: 10.1038/srep38912 27966590 PMC5155238

[54] Schrack JA Zipunnikov V Simonsick EM Studenski S Ferrucci L. Rising energetic cost of walking predicts gait speed decline with aging. J Gerontol A Biol Sci Med Sci (2016) 71(7):947–53. doi: 10.1093/gerona/glw002 PMC4906328 26850913

[55] Jansen SW Roelfsema F van der Spoel E Akintola AA Postmus I Ballieux BE. Familial longevity is associated with higher TSH secretion and strong TSH-fT3 relationship. J Clin Endocrinol Metab (2015) 100(10):3806–13. doi: 10.1210/jc.2015-2624 26230295

[56] Rozing MP Houwing-Duistermaat JJ Slagboom PE Beekman M Frölich M de Craen AJM. Familial longevity is associated with decreased thyroid function. J Clin Endocrinol Metab (2010) 95(11):4979–84. doi: 10.1210/jc.2010-0875 20739380

[57] Jansen SW Akintola AA Roelfsema F van der Spoel E Cobbaert CM Ballieux BE. Human longevity is characterised by high thyroid stimulating hormone secretion without altered energy metabolism. Sci Rep (2015) 5:11525. doi: 10.1038/srep11525 26089239 PMC4473605

[58] Atzmon G Barzilai N Surks MI Gabriely I. Genetic predisposition to elevated serum thyrotropin is associated with exceptional longevity. J Clin Endocrinol Metab (2009) 94(12):4768–75. doi: 10.1210/jc.2009-0808 PMC2795660 19837933

[59] Gussekloo J van Exel E de Craen AJ Meinders AE Frölich M Westendorp RG. Thyroid function, activities of daily living and survival in extreme old age: the ‘Leiden 85-plus Study’. Ned Tijdschr Geneeskd (2006) 150(2):90–6. 16440564

[60] Franceschi C Ostan R Mariotti S Monti D Vitale G. The aging thyroid: A reappraisal within the geroscience integrated perspective. Endocr Rev (2019) 40(5):1250–70. doi: 10.1210/er.2018-00170 31074798

[61] Hine C Kim HJ Zhu Y Harputlugil E Longchamp A Souza M. Hypothalamic-pituitary axis regulates hydrogen sulfide production. Cell Metab (2017) 25(6):1320–1333.e5. doi: 10.1016/j.cmet.2017.05.003 28591635 PMC5722247

[62] Umezu T Kita T Morita M. Dataset on effects of perinatal exposure to propylthiouracil on serum T4, body weight gain, day of eye opening and brain monoamine contents in offspring mice. Data Brief (2020) 28:104900. doi: 10.1016/j.dib.2019.104900 31872013 PMC6909211

[63] Bowers J Terrien J Clerget-Froidevaux MS Gothié JD Rozing MP Westendorp RGJ. Thyroid hormone signaling and homeostasis during aging. Endocr Rev (2013) 34(4):556–89. doi: 10.1210/er.2012-1056 23696256

[64] van den Beld AW Visser TJ Feelders RA Grobbee DE Lamberts SW. Thyroid hormone concentrations, disease, physical function, and mortality in elderly men. J Clin Endocrinol Metab (2005) 90(12):6403–9. doi: 10.1210/jc.2005-0872 16174720

[65] Spadaro O Youm Y Shchukina I Ryu S Sidorov S Ravussin A. Caloric restriction in humans reveals immunometabolic regulators of health span. Science (2022) 375(6581):671–7. doi: 10.1126/science.abg7292 PMC10061495 35143297

[66] Weyh C Krüger K Strasser B. Physical activity and diet shape the immune system during aging. Nutrients (2020) 12(3):4–10. doi: 10.3390/nu12030622 PMC7146449 32121049

[67] Venditti P Di Meo S. Thyroid hormone-induced oxidative stress. Cell Mol Life Sci (2006) 63(4):414–34. doi: 10.1007/s00018-005-5457-9 PMC11136030 16389448

[68] Wieland DR Wieland JR Wang H Chen Y-H Lin C-H Wang J-J. Thyroid disorders and dementia risk: A nationwide population-based case-control study. Neurology (2022) 99(7):e679–87. doi: 10.1212/WNL.0000000000200740 35794019

[69] Abbey EJ McGready J Oh E Simonsick EM Mammen J. Thyroid hormone use and overuse in dementia: Results from the Health, Aging and Body Composition Study. J Am Geriatr Soc (2022) 70(11):3308–11. doi: 10.1111/jgs.17961 PMC9669113 35866295

[70] Perez-Zepeda MU Almeda-Valdes P Fernandez-Villa JM Gomez-Arteaga RC Borda MG Cesari M. Thyroid stimulating hormone levels and geriatric syndromes: secondary nested case-control study of the Mexican Health and Aging Study. Eur Geriatr Med (2022) 13(1):139–45. doi: 10.1007/s41999-021-00564-7 PMC8863630 34601711

[71] Biondi B Cooper DS. The clinical significance of subclinical thyroid dysfunction. Endocr Rev (2008) 29(1):76–131. doi: 10.1210/er.2006-0043 17991805

[72] Rossouw JE Anderson GL Prentice RL LaCroix AZ Kooperberg C Stefanick ML. Risks and benefits of estrogen plus progestin in healthy postmenopausal women: principal results From the Women’s Health Initiative randomized controlled trial. JAMA (2002) 288(3):321–33. doi: 10.1001/jama.288.3.321 12117397

[73] Cooke PS Nanjappa MK Ko C Prins GS Hess RA. Estrogens in male physiology. Physiol Rev (2017) 97(3):995–1043. doi: 10.1152/physrev.00018.2016 28539434 PMC6151497

[74] Gold EB Colvin A Avis N Bromberger J Greendale GA Powell L. Longitudinal analysis of the association between vasomotor symptoms and race/ethnicity across the menopausal transition: study of women’s health across the nation. Am J Public Health (2006) 96(7):1226–35. doi: 10.2105/AJPH.2005.066936 PMC1483882 16735636

[75] Freeman EW Sammel MD Lin H Gracia CR Pien GW Nelson DB. Symptoms associated with menopausal transition and reproductive hormones in midlife women. Obstet Gynecol (2007) 110(2 Pt 1):230–40. doi: 10.1097/01.AOG.0000270153.59102.40 17666595

[76] Kravitz HM Janssen I Bromberger JT Matthews KA Hall MH Ruppert K. Sleep trajectories before and after the final menstrual period in the study of women’s health across the nation (SWAN). Curr Sleep Med Rep (2017) 3(3):235–50. doi: 10.1007/s40675-017-0084-1 PMC5604858 28944165

[77] Lamar M Resnick SM Zonderman AB. Longitudinal changes in verbal memory in older adults: distinguishing the effects of age from repeat testing. Neurology (2003) 60(1):82–6. doi: 10.1212/WNL.60.1.82 12525723

[78] Salpeter SR Cheng J Thabane L Buckley NS Salpeter EE. Bayesian meta-analysis of hormone therapy and mortality in younger postmenopausal women. Am J Med (2009) 122(11):1016–1022.e1. doi: 10.1016/j.amjmed.2009.05.021 19854329

[79] Castanho VS Nakamura RT Pinto-Neto AM Faria EC. Postmenopausal therapy reduces catalase activity and attenuates cardiovascular risk. Arq Bras Cardiol (2012) 99(5):1008–14. doi: 10.1590/S0066-782X2012005000097 23108643

[80] De Franciscis P Mainini G Labriola D Leo S Santangelo F Luisi A. Low-dose estrogen and drospirenone combination: effects on metabolism and endothelial function in postmenopausal women with metabolic syndrome. Clin Exp Obstet Gynecol (2013) 40(2):233–5. 23971246

[81] Manolagas SC Kousteni S Jilka RL. Sex steroids and bone. Recent Prog Horm Res (2002) 57:385–409. doi: 10.1210/rp.57.1.385 12017554

[82] Xiang H Zhang J Lin C Zhang L Liu B Ouyang L. Targeting autophagy-related protein kinases for potential therapeutic purpose. Acta Pharm Sin B (2020) 10(4):569–81. doi: 10.1016/j.apsb.2019.10.003 PMC7161711 32322463

[83] Khosla S Melton LJ 3rd Riggs BL. The unitary model for estrogen deficiency and the pathogenesis of osteoporosis: is a revision needed. J Bone Miner Res (2011) 26(3):441–51. doi: 10.1002/jbmr.262 PMC3179298 20928874

[84] Krum SA Chang J Miranda-Carboni G Wang CY. Novel functions for NFκB: inhibition of bone formation. Nat Rev Rheumatol (2010) 6(10):607–11. doi: 10.1038/nrrheum.2010.133 PMC3078572 20703218

[85] Krum SA Miranda-Carboni GA Hauschka PV Carroll JS Lane TF Freedman LP. Estrogen protects bone by inducing Fas ligand in osteoblasts to regulate osteoclast survival. EMBO J (2008) 27(3):535–45. doi: 10.1038/sj.emboj.7601984 PMC2241656 18219273

[86] Wend K Wend P Krum SA. Tissue-specific effects of loss of estrogen during menopause and aging. Front Endocrinol (Lausanne) (2012) 3:19. doi: 10.3389/fendo.2012.00019 22654856 PMC3356020

[87] Liu F Day M Muñiz LC Bitran D Arias R Revilla-Sanchez R. Activation of estrogen receptor-beta regulates hippocampal synaptic plasticity and improves memory. Nat Neurosci (2008) 11(3):334–43. doi: 10.1038/nn2057 18297067

[88] Spencer-Segal JL Tsuda MC Mattei L Waters EM Romeo RD Milner TA. Estradiol acts *via* estrogen receptors alpha and beta on pathways important for synaptic plasticity in the mouse hippocampal formation. Neuroscience (2012) 202:131–46. doi: 10.1016/j.neuroscience.2011.11.035 PMC3505616 22133892

[89] Boulware MI Heisler JD Frick KM. The memory-enhancing effects of hippocampal estrogen receptor activation involve metabotropic glutamate receptor signaling. J Neurosci (2013) 33(38):15184–94. doi: 10.1523/JNEUROSCI.1716-13.2013 PMC6618419 24048848

[90] Phan A Lancaster KE Armstrong JN MacLusky NJ Choleris E. Rapid effects of estrogen receptor α and β selective agonists on learning and dendritic spines in female mice. Endocrinology (2011) 152(4):1492–502. doi: 10.1210/en.2010-1273 21285321

[91] Barron AM Pike CJ. Sex hormones, aging, and Alzheimer’s disease. Front Biosci (Elite Ed) (2012) 4:976–97. doi: 10.2741/E434 PMC3511049 22201929

[92] Jacobs EG Kroenke C Lin J Epel ES Kenna HA Blackburn EH. Accelerated cell aging in female APOE-ϵ4 carriers: implications for hormone therapy use. PloS One (2013) 8(2):e54713. doi: 10.1371/journal.pone.0054713 23418430 PMC3572118

[93] Supakul S Okano H Maeda S. Utilization of human induced pluripotent stem cells-derived *in vitro* models for the future study of sex differences in alzheimer’s disease. Front Aging Neurosci (2021) 13:768948. doi: 10.3389/fnagi.2021.768948 34803659 PMC8599796

[94] Revankar CM Cimino DF Sklar LA Arterburn JB Prossnitz ER. A transmembrane intracellular estrogen receptor mediates rapid cell signaling. Science (2005) 307(5715):1625–30. doi: 10.1126/science.1106943 15705806

[95] Gould E Woolley CS Frankfurt M McEwen BS. Gonadal steroids regulate dendritic spine density in hippocampal pyramidal cells in adulthood. J Neurosci (1990) 10(4):1286–91. doi: 10.1523/JNEUROSCI.10-04-01286.1990 PMC6570209 2329377

[96] Hara Y Park CS Janssen WG Roberts MT Morrison JH Rapp PR. Synaptic correlates of memory and menopause in the hippocampal dentate gyrus in rhesus monkeys. Neurobiol Aging (2012) 33(2):421.e17–28. doi: 10.1016/j.neurobiolaging.2010.09.014 PMC3031995 21030115

[97] Hao J Rapp PR Leffler AE Leffler SR Janssen WGM Lou W. Estrogen alters spine number and morphology in prefrontal cortex of aged female rhesus monkeys. J Neurosci (2006) 26(9):2571–8. doi: 10.1523/JNEUROSCI.3440-05.2006 PMC6793646 16510735

[98] Hara Y Yuk F Puri R Janssen WG Rapp PR Morrison JH. Estrogen restores multisynaptic boutons in the dorsolateral prefrontal cortex while promoting working memory in aged rhesus monkeys. J Neurosci (2016) 36(3):901–10. doi: 10.1523/JNEUROSCI.3480-13.2016 PMC4719022 26791219

[99] Bachmann GA Schaefers M Uddin A Utian WH. Microdose transdermal estrogen therapy for relief of vulvovaginal symptoms in postmenopausal women. Menopause (2009) 16(5):877–82. doi: 10.1097/gme.0b013e3181a15606 19458560

[100] Hurh YJ Chen ZH Na HK Han SY Surh YJ. 2-Hydroxyestradiol induces oxidative DNA damage and apoptosis in human mammary epithelial cells. J Toxicol Environ Health A (2004) 67(23-24):1939–53. doi: 10.1080/15287390490514598 15513894

[101] Csekes E Račková L. Skin aging, cellular senescence and natural polyphenols. Int J Mol Sci (2021) 22(23):2–10. doi: 10.3390/ijms222312641 PMC8657738 34884444

[102] Gu Y Han J Jiang C Zhang Y. Biomarkers, oxidative stress and autophagy in skin aging. Ageing Res Rev (2020) 59:101036. doi: 10.1016/j.arr.2020.101036 32105850

[103] Valcavi R Zini M Portioli I. Thyroid hormones and growth hormone secretion. J Endocrinol Invest (1992) 15(4):313–30. doi: 10.1007/BF03348744 1512425

[104] Brown-Borg HM. Hormonal control of aging in rodents: the somatotropic axis. Mol Cell Endocrinol (2009) 299(1):64–71. doi: 10.1016/j.mce.2008.07.001 18674587 PMC4390024

[105] Brown-Borg HM. Hormonal regulation of longevity in mammals. Ageing Res Rev (2007) 6(1):28–45. doi: 10.1016/j.arr.2007.02.005 17360245 PMC1978093

[106] Wiesenborn DS Ayala JE King E Masternak MM. Insulin sensitivity in long-living Ames dwarf mice. Age (Dordr) (2014) 36(5):9709. doi: 10.1007/s11357-014-9709-1 25163655 PMC4453940

[107] Russell SJ Kahn CR. Endocrine regulation of ageing. Nat Rev Mol Cell Biol (2007) 8(9):681–91. doi: 10.1038/nrm2234 17684529

[108] Fahy GM Brooke RT Watson JP Good Z Vasanawala SS Maecker H. Reversal of epigenetic aging and immunosenescent trends in humans. Aging Cell (2019) 18(6):e13028. doi: 10.1111/acel.13028 31496122 PMC6826138

[109] Romond K Alam M Kravets S de Sisternes L Leng T Lim JI. Imaging and artificial intelligence for progression of age-related macular degeneration. Exp Biol Med (Maywood) (2021) 246(20):2159–69. doi: 10.1177/15353702211031547 PMC8718252 34404252

[110] Grzybowski A Brona P Lim G Ruamviboonsuk P Tan GSW Abramoff M. Artificial intelligence for diabetic retinopathy screening: a review. Eye (Lond) (2020) 34(3):451–60. doi: 10.1038/s41433-019-0566-0 PMC7055592 31488886

[111] Zhavoronkov A Mamoshina P Vanhaelen Q Scheibye-Knudsen M Moskalev A Aliper A. Artificial intelligence for aging and longevity research: Recent advances and perspectives. Ageing Res Rev (2019) 49:49–66. doi: 10.1016/j.arr.2018.11.003 30472217

[112] Graham S Depp C Lee EE Nebeker C Tu X Kim H-C. Artificial intelligence for mental health and mental illnesses: an overview. Curr Psychiatry Rep (2019) 21(11):116. doi: 10.1007/s11920-019-1094-0 31701320 PMC7274446

